# Distance-Dependent Multimodal Image Registration for Agriculture Tasks

**DOI:** 10.3390/s150820845

**Published:** 2015-08-21

**Authors:** Ron Berenstein, Marko Hočevar, Tone Godeša, Yael Edan, Ohad Ben-Shahar

**Affiliations:** 1 Department of Industrial Engineering and Management, Ben-Gurion University of the Negev, Beer-Sheva 8410501, Israel; E-Mail: yael@bgu.ac.il; 2 Faculty of Mechanical Engineering, University of Ljubljana, Aškerčeva 6, Ljubljana 1000, Slovenia; E-Mails: marko.hocevar@fs.uni-lj.si (M.H.); tone.godesa@kis.si (T.G.); 3 Department of Computer Science, Ben-Gurion University of the Negev, Beer-Sheva 8410501, Israel; E-Mail: ben-shahar@cs.bgu.ac.il

**Keywords:** sensor registration, control points, artificial control points, sensor fusion

## Abstract

Image registration is the process of aligning two or more images of the same scene taken at different times; from different viewpoints; and/or by different sensors. This research focuses on developing a practical method for automatic image registration for agricultural systems that use multimodal sensory systems and operate in natural environments. While not limited to any particular modalities; here we focus on systems with visual and thermal sensory inputs. Our approach is based on pre-calibrating a distance-dependent transformation matrix (DDTM) between the sensors; and representing it in a compact way by regressing the distance-dependent coefficients as distance-dependent functions. The DDTM is measured by calculating a projective transformation matrix for varying distances between the sensors and possible targets. To do so we designed a unique experimental setup including unique Artificial Control Points (ACPs) and their detection algorithms for the two sensors. We demonstrate the utility of our approach using different experiments and evaluation criteria.

## 1. Introduction

Implementing accurate, selective and economical agricultural robots for various agricultural and horticultural tasks is one of the main goals of precision agriculture (see Table 1). A critical task within this field is the detection of natural objects, a particularly difficult challenge due to the inherent unconstrained environments and the significant variability in all object properties such as color, shape, size, texture, and reflectance properties. Moreover, the highly unstructured scenes are often characterized by large degree of uncertainty, changing illumination and shadow conditions, and severe occlusions. Combined with the sheer complexity of the typical unstructured agricultural scene, it is clear why this domain is considered one of the ultimate challenges of sensory systems, machine vision systems in particular [1].

State-of-the-art fruit detection systems commonly combine several detection sensors and algorithms using sensor fusion techniques to achieve better detection rates (Table 1). Often, the detection module constitutes a single RGB camera combined with one or more complementary sensors such as thermal, infra-red, laser scanner, hyper-spectral, or time of flight (see Table 1 for many examples). To do so successfully, the first mandatory step in fusing the data from the different sensors is the registration of their images. This registration is the focus of our work. While nothing in our approach is tailored to any particular sensor modalities, our case study is the combination of visual and thermal sensors. Indeed, how fruit detection rates can be improved by fusing these particular sensors compared to each of them alone was already demonstrated in the past, for example for the detection of oranges [2].

Thermal imaging allows for finding fruit whose color, potentially an indicative cue, is very sensitive to illumination conditions. Leaves, unlike fruits, accumulate less heat and emit it for a shorter time. However, since the thermal response is sensitive to the sunlight illumination and heat accumulation, fruit on different parts of the tree might respond differently [1,2,3]. In such cases combining thermal and RGB imaging could be useful for fruit detection and harvesting, under the assumption that a suitable registration procedure is provided.

**Table 1:** Examples of various precision agriculture topics of research in the last three decades.

Task	Sensors	Description of Research	Reference
Target detection	RGB, spectral	survey on fruit detection using computer vision	[4]
	RGB camera	detection of grape clusters	[5]
	Thermal vision	detection of oranges using thermal imagery	[6]
	RGB camera + thermal camera	detection of oranges using RGB camera and thermal camera and conducting fusion between	[2]
Precision spraying	RGB camera	selective sprayer for weed control using machine vision, real time controller and controllable spraying system	[7]
	CCD camera	developed a tree crown recognition and smart spraying system	[8]
	BW camera + ultrasonic	speed spraying using fuzzy logic control of machine vision and ultrasonic sensors	[9]
	Ultrasonic	spraying robot for vine production	[10]
Robotic harvesting	Stereo vision	apple harvester robot	[11]
	Color camera (HSV)	robot for greenhouse operations	[12]
	Color camera	apple harvester robot	[13]
	2 wavelengths of color camera	strawberry harvester robot	[14]

**Figure 1:**
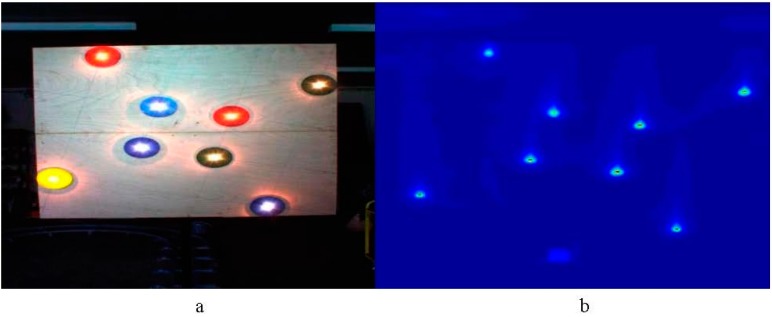
Same scene acquired by different sensors. (**a**) Image from RGB camera; (**b**) Image from thermal camera.

Image registration is the process of overlaying (*i.e.*, transforming into the same coordinate system) two or more images of the same scene taken at different times, from different viewpoints, and/or by different sensors [15]. Registration processes are usually divided into the following steps: (i) **Feature detection**—detects (manually or automatically) the position of distinctive objects in the image, also known as Control Points (CPs). While such features are often the starting point for the registration process [16,17,18,19], in many cases they are replaced with pixel patches that provide distinct appearance [20,21,22]; (ii) **Feature matching**—establishes correspondence between the CPs of the different sensors. For example, in Figure 1 we may wish to correspond the yellow disk in the visual image with the left most point in the thermal image; (iii) **Transformation model estimation**—determines the type of image transformation according to prior information (or lack thereof) regarding the acquisition process; (iv) **Image transformation**—transforms the two images into a common coordinate system (often by transforming one into the other).

Applying automatic image registration for systems that use visual and thermal images is difficult due to the different methods employed to calculate the CPs for each type of sensor and the lack of natural correspondence between their CPs [23]. Most papers dealing with visual and thermal image fusion and registration [23,24] perform this correspondence manually (*i.e.*, use a human to mark corresponding CPs in the visual and thermal images). One exception is Istenic *et al*. [25] who presented an automatic method for visual and thermal image registration. Their approach uses edge detection and Hough transform to detect linear lines in both images and to compute CPs for the registration process. However, since this approach is based on straight linear segments, it is limited only to images that consist of objects with straight lines [25], which are not prevailing in the unstructured agriculture environment, another method must be developed. Introducing landmarks manually for thermal and visible image registration proved successful for aligning static medical images [26,27] and for fruit registration (e.g., [2]).

Among the mentioned papers [23,24,26,27] require manual CP selection. The procedure for Istenic *et al*. [25] is automatic, however their scenes were much more structured.

The goal of this work is to introduce a new approach for highly accurate registration of thermal and color images.

The novelty of the manuscript is that it offers a new registration method suitable for unstructured environments with long intervals of sensing ranges. It introduces a new approach for highly accurate registration of thermal and color images. The registration approach is based on the computation of a “dynamic” transformation matrix (TM) in which each element is a function of the distance from the object in the image. In the field this distance can be measured by a range sensor. In this paper, we demonstrate the utility of our approach on a robotic sprayer equipped with an RGB and thermal camera, as well as a laser scanner. Within the interval, the method offers compact representation of multiple (or infinite, if one considers the continuous range) registration transformations. Thanks to the regression algorithm, the procedure permits registration at distances for which the sensors were not calibrated for. For completeness we also present the ACP design and algorithm for ACP detection, although this is not the core of the paper.

## 2. Methods

### 2.1. Image Registration Model

The core of the registration process is to find TM that transforms pixels in one image to the other. Formally,

(1) $$\left[\begin{array}{c} x\prime \\ y\prime \\ 1 \end{array}\right]=TM\cdot \left[\begin{array}{c} x\\ y\\ 1 \end{array}\right];\;\;\;\;\;TM=\left[\begin{array}{ccc} h_{00} & h_{01} & h_{02}\\ h_{10} & h_{11} & h_{12}\\ h_{20} & h_{21} & h_{22} \end{array}\right]$$

where *x*' and *y*' is the registered pixel coordinates and *x*, *y* is the original pixel coordinates. To be general enough, here we assume that the transformation between the multimodal images is projective, an eight DOF (Degree Of Freedom) mapping which contributes to the registration accuracy [28].

The projective transformation is a 3 × 3 homogeneous matrix (TM). Since it has eight DOFs, a linear system of rank eight is required in order to determine the value for each of the TM elements. Each CP (identified by its two coordinates) can contribute two constraints by applying Equation (1) and rearranging as follows

(2) $$\begin{array}{l} x\prime =\frac{h_{00}x+h_{01}y+h_{02}}{h_{20}x+h_{21}y+h_{22}}\;\;\;\;\;\;\;y\prime =\frac{h_{10}x+h_{11}y+h_{12}}{h_{20}x+h_{21}y+h_{22}}\\ h_{00}x+h_{01}y+h_{02}-h_{20}xx\prime -h_{21}yx\prime -h_{22}x\prime =0\\ h_{10}x+h_{11}y+h_{12}-h_{20}xy\prime -h_{21}yy\prime -h_{22}y\prime =0 \end{array}$$

By repeating this procedure with at least four CP, a full linear system can be created and solved in order to determine the values of the TM.

(3) $$\left[\begin{array}{cccccccc} x_{1} & y_{1} & 1 & 0 & 0 & 0 & -x_{1}\prime \cdot x_{1} & -x_{1}\prime \cdot y_{1}\\ 0 & 0 & 0 & x_{1} & y_{1} & 1 & -y_{1}\prime \cdot x_{1} & -y_{1}\prime \cdot y_{1}\\ & & & & \vdots & & & \\ x_{n} & y_{n} & 1 & 0 & 0 & 0 & -x_{n}\prime \cdot x_{n} & -x_{n}\prime \cdot y_{n}\\ 0 & 0 & 0 & x_{n} & y_{n} & 1 & -y_{n}\prime \cdot x_{n} & -y_{n}\prime \cdot y_{n} \end{array}\right]\cdot \left[\begin{array}{c} h_{00}\\ h_{01}\\ h_{02}\\ h_{10}\\ h_{11}\\ h_{12}\\ h_{20}\\ h_{21} \end{array}\right]=\left[\begin{array}{c} x_{1}\prime \\ y_{1}\prime \\ \begin{array}{l} \vdots \end{array}\\ x_{n}\prime \\ y_{n}\prime \end{array}\right]$$

Four CPs is the minimum required to solve the projective transformation matrix (TM) but using more CPs can provide resistance to noise and contribute to the registration accuracy. In this case the system in Equation (3) becomes overdetermined and its least squares optimal solution can be found with singular value decomposition (SVD).

The TM just described provides the registration between two given images, but cannot align arbitrary two images captured in the field where CPs are not available in real time. For this we expand the process to support image pairs of arbitrarily distant objects via pre-calibrated registration. The base of this approach is the Distance Dependent Transformation Matrix (DDTM) (*TM(D)*)—A TM that depends on distance *D*. We construct this matrix function as follows

**Figure 2:**
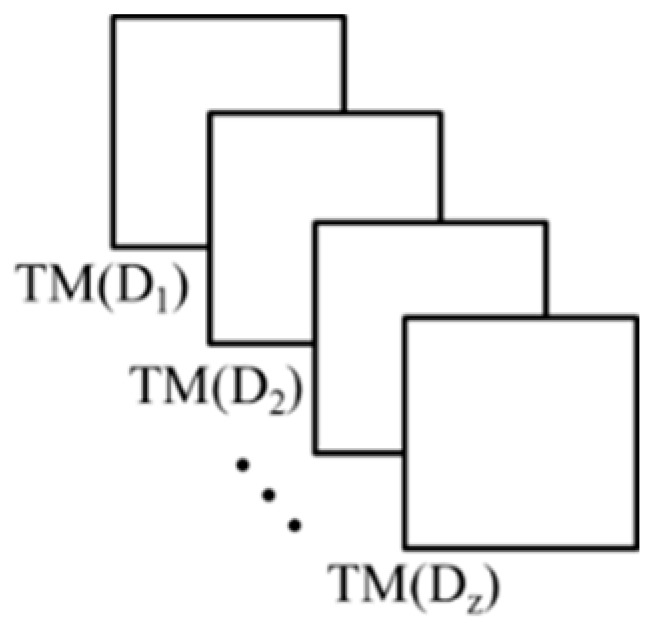
TM collection.

(i) Capture a scene with varying distances between the sensors and the CPs,
(ii) Calculate the TM for each scene with its corresponding distance,
(iii) Construct a collection of all the TM calculated (Figure 2),
(iv) Collect the corresponding values from each element of each matrix, use them as samples of a distance-dependent function, and perform a regression *R* with these samples to obtain a functional representation of the element
  (4) $$\text{DDTM}\;\;\;\;{\left(D\right)}_{\left(m,n\right)}=R\;\;\left(TM{\left(D_{1}\right)}_{\;(m,n)},TM{\left(D_{2}\right)}_{\;(m,n)},...,TM{\left(D_{z}\right)}_{\left(m,n\right)}\right)$$
  where *m* and *n* are the row and column indices of the TM respectively,
(v) Collect all functions to construct the DDTM.

Using Equation (1) while defining the TM component as the newly defined DDTM (Equation (5)), each new pixel’s coordinates will be calculated according to its distance D (which is assumed measured).

(5) $$DDTM\left(D\right)=\left[\begin{array}{ccc} DDTM{\left(D\right)}_{\left(0,0\right)} & DDTM{\left(D\right)}_{\left(0,1\right)} & DDTM{\left(D\right)}_{\left(0,2\right)}\\ DDTM{\left(D\right)}_{\left(1,0\right)} & DDTM{\left(D\right)}_{\left(1,1\right)} & DDTM{\left(D\right)}_{\left(1,2\right)}\\ DDTM{\left(D\right)}_{\left(2,0\right)} & DDTM{\left(D\right)}_{\left(2,1\right)} & DDTM{\left(D\right)}_{\left(2,2\right)} \end{array}\right]$$

The above equation is the core of a very compact registration of thermal and RGB camera images depending on the distance, which will be in our case measured with a laser scanner.

### 2.2. Target Rail and Sensor Position

**Figure 3:**
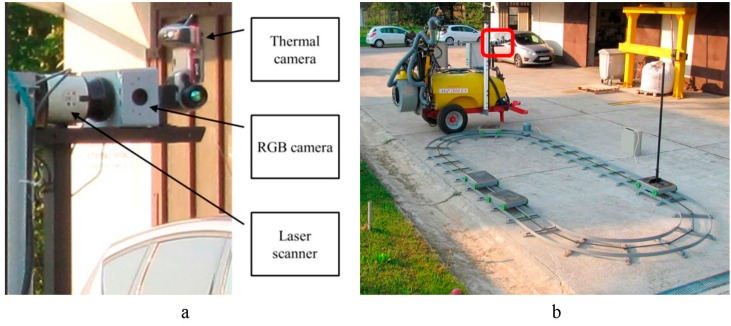
Selective sprayer. (**a**) sensors array; (**b**) overall sprayer with rail (red square, sprayer sensors).

The experimental system included two main elements: sensor array (Figure 3a) and a circular rail with mobile rail-cart (Figure 3b). The sensor array contained three sensors, including a RGB camera (Flea2 FL2-08S2C with resolution of 768 × 1032 and 45° wide angle lens, acquisition frequency 30 FPR), a thermal camera (Flir T425 with resolution of 240 × 320 and 45° IR wide angle lens, acquisition frequency 9 FPR), and a laser scanner (Sick LMS111 with scanning angle of 270° and resolution of 0.5°, scanning frequency 50 Hz). The sensors were mounted to the sprayer disabling any relative movement among sensors during all experiments. The second experimental element is a circular rail (width 6.4 m/length 2.4 m) with a mobile rail-cart (Figure 3b) able to move along the rail line. The rail-cart position/velocity can be controlled manually or by using an AC electric motor with frequency inverter regulation. Several types of targets can be fixed on top of the rail-cart such as artificial targets or real-world target (live trees). Data from all three sensors was acquired simultaneously using a personal computer and stored for post analysis.

### 2.3. Artificial Control Points

**Figure 4:**
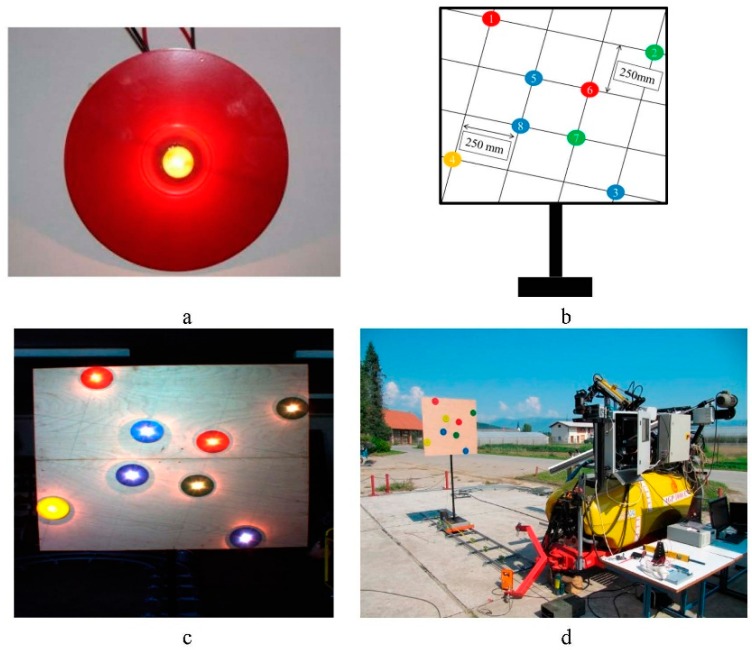
ACPs (**a**) single target with an incandescent light bulb in the middle; (**b**) ACPs setup; (**c**) ACPs mounting plate; (**d**) DDTM evaluation setup.

To construct the DDTM, the collection of TMs that register a preconfigured pair of sensors had to be derived from experimental data. To do so, a unique type of artificial control point (ACP) was designed with the goal to be easily detected by the two sensors, the RGB camera and the thermal camera. Each of our ACPs is composed of two elements, a colored disk (120 mm diameter) with 10 W incandescent light bulb fixed at the disk center (Figure 4a,c). The= disks were colored differently to provide better visualization. While not used here, in future applications these different colors can be used to associate unique identities to the markers in order to facilitate more elaborate inference about the spatial organization of the ACPs.

In order to calculate a reliable DDTM in a real world scene, eight ACPs were mounted to a flat plate (1 × 1 m) as shown in Figure 4b. The plate dimensions and the ACPs arrangement within the plate were chosen such that at the minimum operational distance between the plate and the sensors array (170 cm) the ACPs will cover most of the image area and provide maximal sensitivity in their position. In order to evaluate the DDTM, the ACPs flat plate was mounted to the rail-cart with the ability to move while the plate remains perpendicular to the sensors array (Figure 4d).

**Figure 5:**
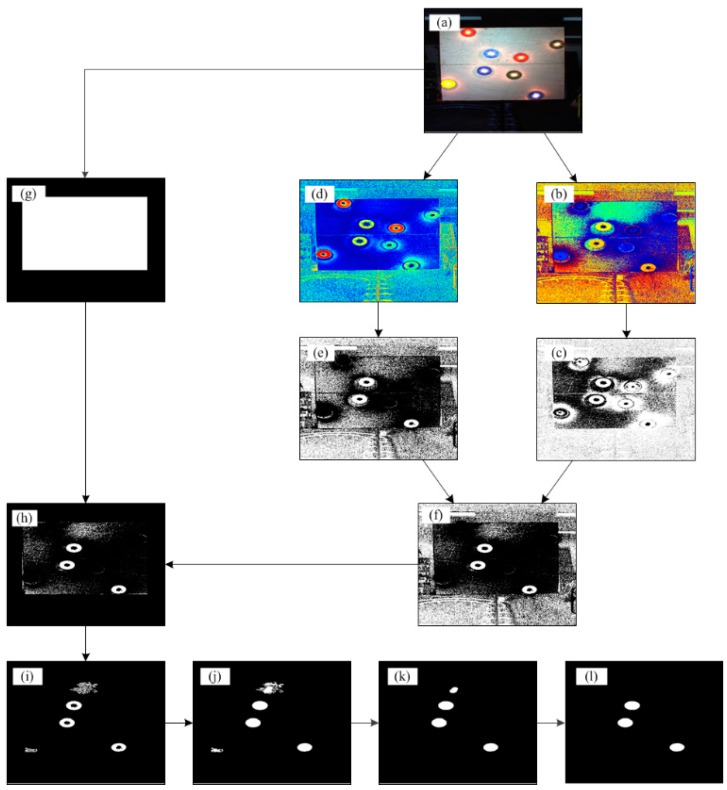
Blue colored disk detection algorithm.

The colored disk detection algorithm was designed to detect and localize the ACPs in the color image and is based on commonly used machine vision procedures. The algorithm was implemented using Matlab software and Matlab image processing toolbox and was based on color thresholding (according to the specific disk). Figure 5 shows the detection of three blue disks in the image, but the algorithm is easily modified according to the desired target color. More specifically, the algorithm works as follows:

(i) Capture input RGB image (Figure 5a),
(ii) Convert the RGB image to HSV representation and isolate the hue and saturation channels (Figure 5b,d where (b) is the Hue and (d) is the saturation),
(iii) Threshold the hue and the saturation (thresholds were set according to the detected color) (Figure 5c,e),
(iv) Merge (logical OR) the resulted binary images (step iii, Figure 5f),
(v) Isolate the ACPs plate using the RGB image (Figure 5g) (the ACPs plate is brighter relative to the environment and by applying threshold on the R,G,B channels the ACPs plate can be identified and isolate),
(vi) Unite the binary ACP plate (Figure 5g) with the binary image resulted in step iv (Figure 5f),
(vii) Remove small clusters (<500) of pixels that considered as noise (using Matlab command *bwareaopen*) (Figure 5i),
(viii) Fill holes in the image using morphological operations (using Matlab command *imfill*) (Figure 5j),
(ix) Apply erosion followed by dilation using disk mask (*r* = 15 pixel) (Figure 5k). Using the disk shape mask, this step helps to remove small, non-ellipse shapes,
(x) Filter remaining identification errors by searching saturated pixels in the shape center (expected due to the light bulb) (Figure 5l).

The detection of ACPs in the thermal images, *i.e.*, the detection of the light bulbs, followed the following steps:

(i) Capture a thermal image,
(ii) Threshold the image for high temperature values,
(iii) Remove small clusters (<10) of pixels that considered as noise (using Matlab command *bwareaopen*),
(iv) Apply erosion followed by dilation using disk mask (*r* = 5). Using the disk shape mask, this step helps to remove small, non-ellipse shapes.

**Figure 6:**
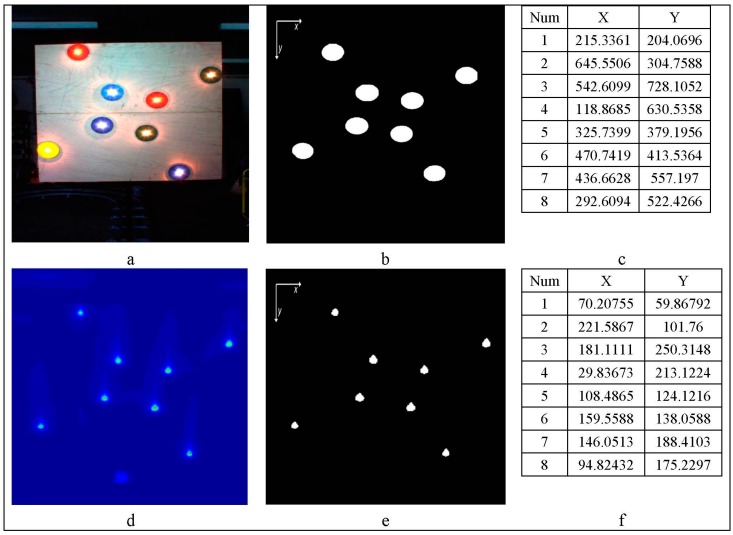
(**a**) visual image; (**b**) RGB ACPs positions, (**c**) RGB ACPs position; (**d**) thermal image; (**e**) thermal ACPs position; (**f**) ACPs position; (**c**,**f**) are sorted according to Figure 4b.

The ACP position in the visual and thermal image was calculated as the center of mass of each ACP position (Figure 6).

### 2.4. ACP Analysis

To validate the ACPs detection algorithms, we compared their detected locations with ground truth position marked by a human operator on a set of 192 points (24 random images, eight points in each image). The distance *d_RGB_* pixels, between the automatically and manually marked ACPs was considered as the detection error and calculated by the standard Euclidean formula

(6) $$\begin{array}{l} d_{RGB}=\sqrt{{\left(x_{RGB}-x_{M\;RGB}\right)}^{2}-{\left(y_{RGB}-y_{M\;RGB}\right)}^{2}}\\ d_{Thermal}=\sqrt{{\left(x_{Thermal}-x_{M\;Thermal}\right)}^{2}-{\left(y_{Thermal}-y_{M\;Thermal}\right)}^{2}} \end{array}$$

where *d_RGB_* is the distance in pixels, *x_RGB_* and *y_RGB_* are the automatically calculated horizontal and vertical coordinates of the visual image respectively, *x_M RGB_* and *y_M RGB_* are the manually marked horizontal and vertical coordinates of the visual image respectively, *x_Thermal_* and *y_Thermal_* are the automatically calculated horizontal and vertical coordinates of the thermal image respectively, *x_M Thermal_* and *y_MThermal_* are the manually marked horizontal and vertical coordinates of the thermal image respectively. Since the visual and thermal cameras have different resolutions, a normalization correction was added according to Equation (7). Needless to say, these constants require adjustment for other cameras and lenses.

(7) $$d_{RGB}=\sqrt{{\left(\left(x_{Thermal}-x_{M\;Thermal}\right)\cdot \frac{1032}{320}\right)}^{2}-{\left(\left(y_{Thermal}-y_{M\;Thermal}\right)\cdot \frac{768}{240}\right)}^{2}}$$

Lens distortions play an important role in the registration procedure. Although distortion can be irregular or follow many patterns, the most commonly encountered distortions are approximately radially symmetric, arising from the symmetry of a photographic lens. This is true for both thermal and visual light lenses. However, results summarized later in Table 3 show that these were not very large and that in accordance h20 and h21 were close to zero.

Table 2 shows that the average error between the automatically and manually marked ACPs is 1.36 pixel and 0.87 pixel for the visual and the thermal image respectively. Comparison between the ACPs detection algorithms shows that the color disk detection algorithm is more accurate than the thermal detection algorithm (for this comparison, normalized thermal data is used).

**Table 2:** Error comparison between manual and automatically ACPs detection.

Sensor	Average	Standard Deviation
	pixel	pixel
RGB	1.36	0.88
Thermal	0.87	0.32
Thermal (normalized)	2.7	1.03

## 3. Experimental Evaluation

With the approach outlined above, experimental evaluation was performed in three steps. First, we constructed the DDTM using measured data. Second, we evaluated the constructed DDTM under easy\simple conditions. Evaluation of performance in real-world conditions is described in the third step which measures performance for different vibrations (pulling tractor, wind) and target registration of oblique objects.

### 3.1. Experiment 1: Estimation of the DDTM

The first experiment goal was to create the *DDTM(D)*. Note that this computation is needed for each multimodal sensory configuration and can be considered as its calibration. Doing so requires sampling of corresponding ACPs, solving their TMs from several distances and then performing regression on the numerical results (as described in ‎Section 2.1).

In order to calculate the *DDTM(D)* in our experiment, a set of 51 scenes were captured from each distance and was used for better estimation of the ACPs position. The 51 scenes were captured sequentially during time frame of ~5.5 s. The scenes were captured by varying the distances between the sensor array and the ACPs plate from 1700 to 3000 mm with intervals of 50 mm resulting in a total of 27 distances, 408 ACPs for each distance, and a total of 1377 images which contributed to the DDTM estimation. These distances were selected because they correspond well to the operational distances of the specific sprayer system we used for evaluation. Each captured scene includes a single visual image from the RGB camera, a single thermal image, and a single laser scan that includes the plate surface.

**Figure 7:**
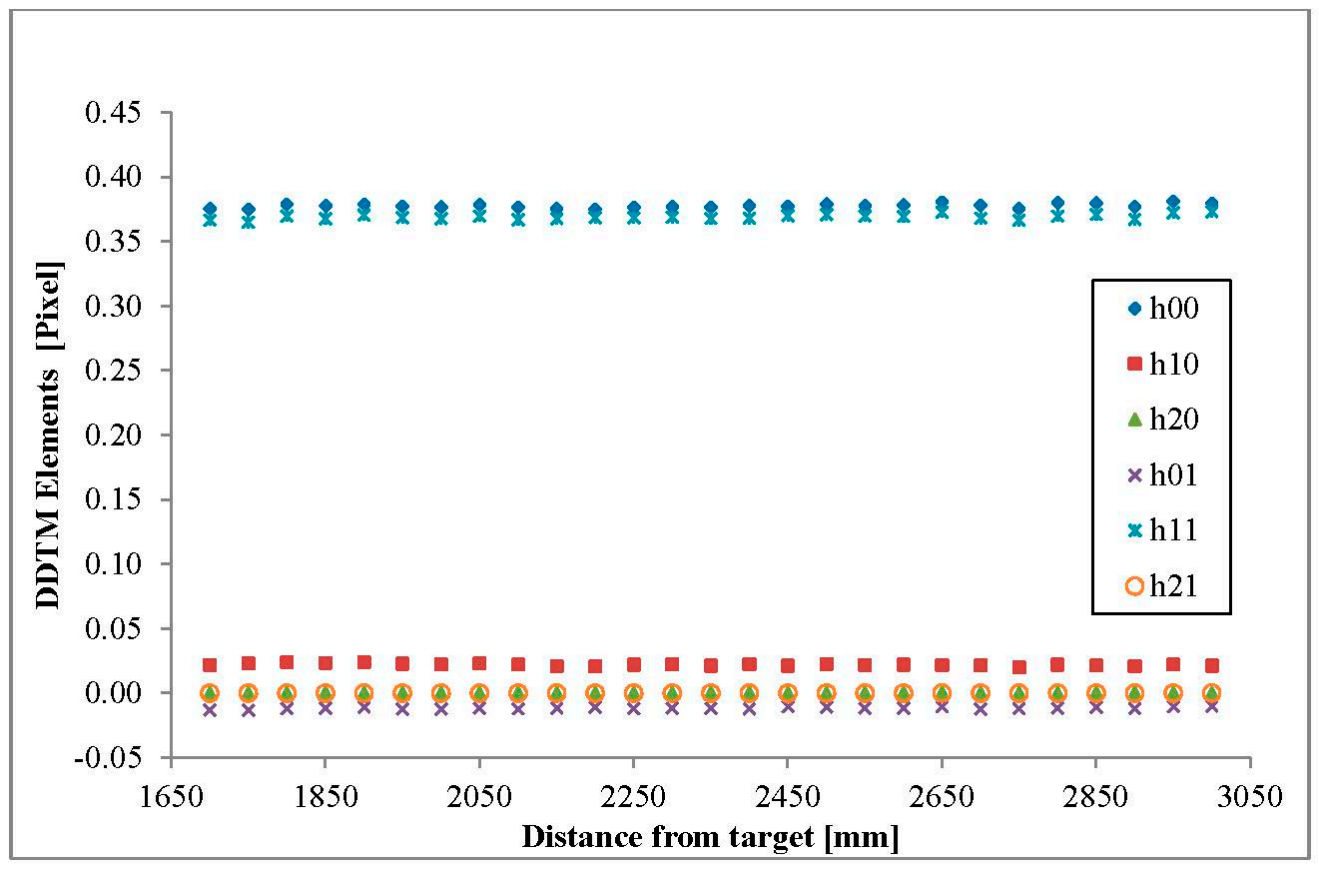
DDTM elements (*h_00_*, *h_10_*, *h_20_*, *h_01_*, *h_11_*, *h_21_*).

**Figure 8:**
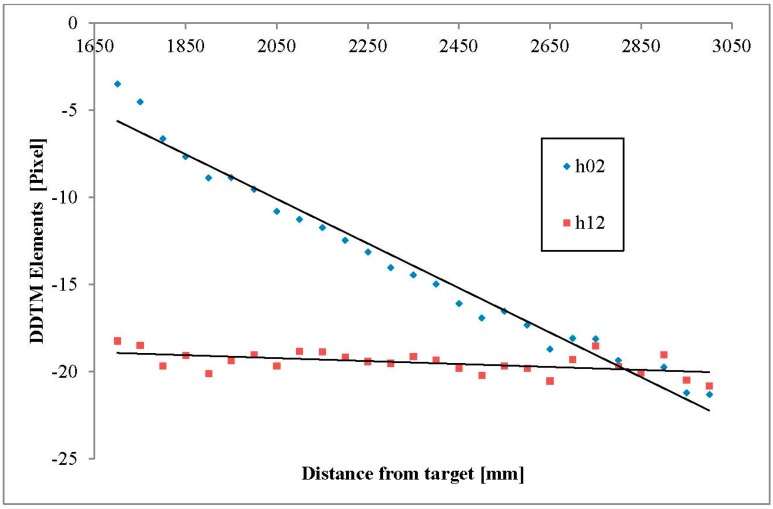
DDTM elements (*h_02_*, *h_12_*).

According to the suggested model in Section 2.1, a linear regression was used on each of the TM elements (Figure 7 and Figure 8 and Table 3). The linear equations of *h_00_*, *h_01_*, *h_10_*, *h_11_*, *h_20_* and *h_21_* were approximated to a constant value (the second linear coefficient) since the slope coefficient was extremely small (Table 3), *i.e.*, only *h_02_* and *h_21_* were affected by the distance between the plate and the sensor array. The *DDTM(D)* calculated for our particular sensor array was therefore presented as follows:

(8) $$DDTM(D)=\left[\begin{array}{ccc} 0.3729 & -0.01408 & -0.012784\cdot D+16.110487\\ 0.025381 & 0.363118 & -0.000845\cdot D-17.503239\\ 0.000090 & -0.000014 & 1 \end{array}\right]$$

where *D* mm is the distance between the sensors array and the target for which registration is performed.

**Table 3:** DDTM regression summary for the sensor configuration used in our experiment.

$DDTM_{\left(m,n\right)}(D)$	Regression Result	Approximation as Constant
h_00_	*f(D)* = 2.15E−06·*D* + 3.72E−01	0.3729
h_01_	*f(D)* = 1.06E−06·*D* − 1.41E−02	−0.014085
h_02_	*f(D)* = −0.012784·*D* − 16.110487	
h_10_	*f(D)* = −1.44E−06·*D* − 2.54E−02	0.025381
h_11_	*f(D)* = 2.44E−06·*D* − 3.63E−01	0.363118
h_12_	*f(D)* = −0.000845·*D* − 17.503239	
h_20_	*f(D)* = −5.56E−09·*D* + 9.00E−05	0.000090
h_21_	*f(D)* = 3.71E−09·*D* − 1.45E−05	−0.000014

Equation (8) and the results summarized in Table 3 indicate that *h_20_* and *h_21_* are close to zero which suggests that the resulting DDTM is an affine TM type with the following shape:

(9) $$affine\;\;\;TM=\left[\begin{array}{cc} A & t\\ 0^{T} & 1 \end{array}\right]$$

where *A* is a 2 × 2 matrix responsible for the rotation and deformation and *t* responsible for the translation [29]. *A* elements in Equation (9) can be decomposed and pose as:

(10) A=R(θ)R(−φ)DR(φ)

where *R(Ɵ)* and *R(Φ)* are rotation by *Ɵ* and deformation by *Φ*, and D is a diagonal scaling matrix:

$$D=\left[\begin{array}{cc} \lambda_{1} & 0\\ 0 & \lambda_{2} \end{array}\right]$$

If the two cameras were perfectly aligned in parallel (horizontal and vertical) then *Ɵ* and *Φ* values would be zeros, resulting in:

(11) $$A=D=\left[\begin{array}{cc} \lambda_{1} & 0\\ 0 & \lambda_{2} \end{array}\right]$$

and the values of λ_1_ and λ_2_ would be the cameras resolution ratios,

$$\begin{array}{l} \lambda_{1}=\frac{320}{1032}=0.31\\ \lambda_{2}=\frac{240}{768}=0.3125 \end{array}$$

But since our sensors also sustained minor misalignment setup (as expected in any field setup), the *Ɵ* and *Φ* are not zeros and the results of *A* are DDTM elements (*h_00_*, *h_01_*, *h_10_*, *h_11_*).

**Figure 9:**
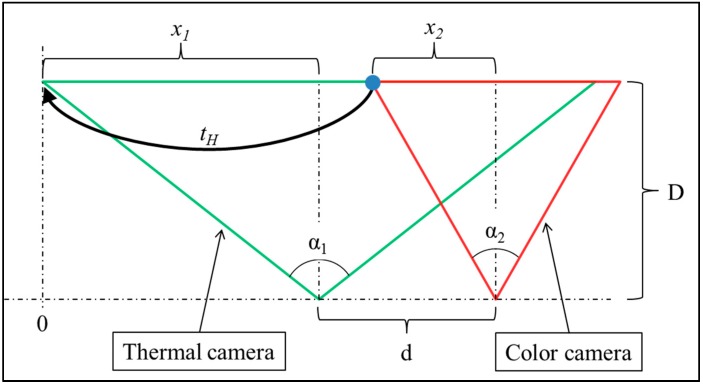
Horizontal translation example.

The translation elements (*h_02_* and *h_12_*) are linear as shown in the following:

Consider the rectified setup shown in Figure 9, where two cameras are horizontally aligned and the distance between their centers (a.k.a. the baseline) is *d*. The thermal and color cameras horizontal fields of view are α_1_ and α_2_, respectively. The distance between the cameras and the target is *D*. In order to horizontally translate (t_H_) the left pixel of the color camera (Figure 9 blue circle) to the corresponding left of the thermal camera, the following should be calculated:

$$\begin{array}{l} t_{H}=x_{1}+d+x_{2}\\ x_{1}=D\cdot \tan\left(\frac{\alpha_{1}}{2}\right)\\ x_{2}=D\cdot \tan\left(\frac{\alpha_{2}}{2}\right)\\ t_{H}=D\cdot \tan\left(\frac{\alpha_{1}}{2}\right)+d+D\cdot \tan\left(\frac{\alpha_{2}}{2}\right)\\ t_{H}=D\cdot \left(\tan\left(\frac{\alpha_{1}}{2}\right)-\tan\left(\frac{\alpha_{2}}{2}\right)\right)+d \end{array}$$

and since α_1_ and α_2_ are constants, *t_H_* is linear. The vertical translation can be shown to be linear in the same calculation procedure.

### 3.2. Experiment 2: DDTM Evaluation, Straight Vertical Plane

Once the DDTM was determined as shown above, we next evaluated it for registration of controlled measured data. In this experiment the ACPs will change their designation and be used as image registration validation points. The ACP RGB coordinate will be mapped to the thermal image. Ideally, by applying the registration, the mapped RGB coordinates should align with the thermal coordinates, while any difference will be considered as a registration error calculated using:

$$\begin{array}{l} x_{e}=\left(x\prime_{C}-x_{T}\right)\cdot \frac{559}{RP_{D}}\\ y_{e}=\left(y_{C}\prime -y_{T}\right)\cdot \frac{559}{RP_{D}}\\ d_{e}=\sqrt{x_{e}^{2}+y_{e}^{2}} \end{array}$$

where *x_e_* and *y_e_* are the errors in *x* and *y* coordinates of the registered color image (*x*'*_C_*, *y_C_*') to the thermal image (*x_T_*, *y_T_*), *d_e_* is the distance error and *RP_D_* is the pixel distance between the red CPs in the thermal image. The error is normalized to millimeter units by multiplying the registration error by the physical distance between CPs 1 and 6 in Figure 4b

$$\sqrt{250^{2}+500^{2}}=559\;(\text{mm})$$

divided by the pixel distance between CPs 1 and 6 detected in the thermal image.

To run the evaluation, we acquired new sets of images of the visual and thermal ACPs at each of the distances used above (*i.e.*, 1700 to 3000 mm with 50 mm intervals between). After acquisition, the visual ACPs were mapped to the thermal ACPs using the DDTM(D) instances. For each distance, 51 scenes were captured. The registration error was calculated for each CPs in the capture scene (51 scenes for each distance) for each of the distances (1700~3000).

**Figure 10:**
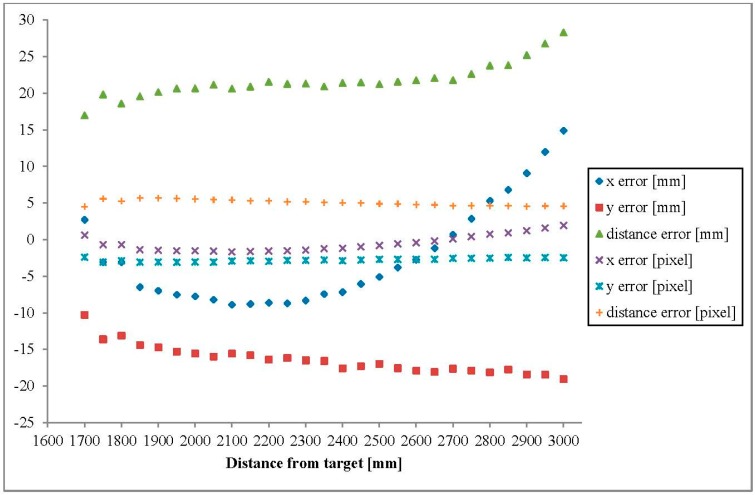
Registration errors while applaying DDTM.

Registration error evaluation (Figure 10) reveals that while considering the error in terms of pixels, the error remains close to constant value ([0, –2] for x, [–2, –3] for y and [3, 5] for the distance error) but when considering the error in terms of physical distance mm the error increases with the distance from the target (*D*). The maximum registration error was no more than 3–5 pixels when using the DDTM(D).

### 3.3. Experiment 3: DDTM Evaluation, Oblique ACPs Plane and Sensor Vibration

The objectives of the third experiment were to simulate more realistic sensory scenarios. We first examined performance when each image pixel is mapped according to the specific distance from the sensors. Second, we evaluated the registration performance while applying vibrations to the sensors unit, a condition imitating the vibrations that a pulling tractor would impose when driving and pulling the sprayer on uneven ground.

**Figure 11:**
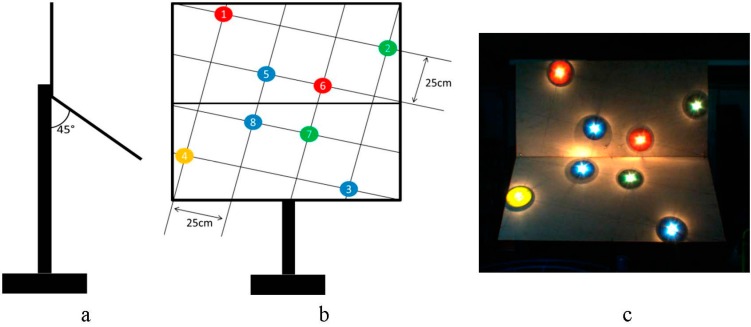
ACPs arrangement. (**a**) side view; (**b**) front view; (**c**) visual image.

The experimental setup included two flat plates connected at 45° (Figure 11a). Four ACPs were attached to each of the plates with a total of 8 ACPs (Figure 11b). The oblique plates were mounted to the rail-cart with the ability to move while the plates remain perpendicular to the sensors array. Figure 11c shows sample of the visual image captured from the RGB camera.

During each experiment, the sprayer was stationary, while the rail cart with ACPs moved perpendicular to the sprayer and sensing unit. Sensing unit vibrations were in the Cartesian space. During the experiment, many Eigen—frequencies of the sprayer, half—filled reservoir, modifications to the sprayer *etc.* resulted in different sprayer (including sensing equipment) vibration levels for each coordinate of Cartesian space and for each vibration frequency setting. Due to a variety of vibration modes and few operating points the vibrations are expressed as total acceleration as shown in Table 4.

**Table 4:** Results of the third experiment.

Vibration Frequency Hz (Swing Arm Rotation Frequency Hz)	Acceleration g	Number of Captured Images	Vertical Plane CP1, CP2, CP5, CP6 pixel		Oblique PlaneCP3, CP4, CP7, CP8 pixel	
			Mean Error	Standard Deviation	Mean Error	Standard Deviation
0	0	118	3.68	0.67	4.02	0.86
2.18	0.011	118	3.34	0.79	4.18	1.20
2.49	0.0577	118	3.29	0.72	4.42	1.21
2.80	0.0595	118	3.38	0.62	3.62	0.98
3.11	0.1117	118	3.07	0.70	3.81	1.04
3.42	0.1941	118	3.38	0.84	3.89	1.19
3.73	0.3467	118	4.07	1.33	4.19	1.51
4.04	0.3252	118	3.91	1.23	4.11	1.43
4.35	0.2433	118	3.45	0.87	3.72	1.32
4.66	0.2596	118	3.74	1.10	3.76	1.13
4.97	0.3575	118	3.55	0.96	4.20	1.33
5.28	0.4519	118	3.68	0.88	4.13	1.20

The vibrations were created using a frequency controlled AC electric motor with an eccentrically attached mass. The electric motor with its eccentrically attached mass was bolted to the sprayer’s sensor array. Induced vibrations caused the entire sprayer to shake. During this experiment, the sprayer tank was filled with 500 liters of water, simulating a typical load. Vibrations were measured using an accelerometer sensor (Phidgets 1043 with an acquisition frequency of 100 Hz) mounted rigidly on the sensing equipment support arm.

During the experiment, 12 electric motor rotational frequencies were evaluated, while the mass and its eccentric attached point remained fixed. The first frequency was set to zero (no vibration) and used as a reference to the vibration measurement. For each frequency, the rail-cart traveled from distance 1700 to 3000 mm and back to 1700 mm along the rail while the scene was captured continuously during the movement.

The mean registration error and the mean standard deviation were calculated for all test cases according to Table 4.

Comparison between the different vibrations shows that the vibration has no effect on the DDTM performance. This can be explained by the high frame rate of the sensors (30 and 9 frames per second for the color and thermal camera respectively).

## 4. Discussion and Conclusions

We have developed and presented a practical method for registration of multimodal sensory rigs for agricultural tasks. Our approach is based on pre-calibrating a distance-dependent TM between the sensors, and representing it in a compact way by regressing the distance-dependent coefficients as distance-dependent functions. In our case these dependencies ended up linear, but more elaborate DDTM may be obtained in more complicated situations.

Error evaluation of x and y coordinates show that while considering the registration error in the pixel space, the error remains relatively constant, but when considering the error in the Euclidean distance space the error increases with the distance between the sensors and the target.

The registration method suggested was developed for use with a one dimensional distance sensor (laser scanner). The method can be easily extended for a two dimensional distance sensor (e.g., Kinect sensor, Time of Flight camera) and gain higher accuracy from using the known distance of each image pixel.

While the presented approach was developed for an agricultural environment application, it can be applied to other applications that require registration of objects at varying distances. The method can be used to register images from all imaging sensors providing the sensors can detect common control points.
